# Specialization in the marketplace for ideas

**DOI:** 10.1371/journal.pone.0293355

**Published:** 2023-10-25

**Authors:** Sirui Wang, Michael Macy, Victor Nee

**Affiliations:** 1 Department of Operations, Information and Decisions, The Wharton School, University of Pennsylvania, Philadelphia, Pennsylvania, United States of America; 2 Department of Information Science, Cornell University, Ithaca, New York, United States of America; 3 Department of Sociology, Cornell University, Ithaca, New York, United States of America; Universidad Complutense de Madrid, SPAIN

## Abstract

Organizations that compete for attention in the marketplace face a strategic decision: whether to target a specialized niche or diversify to reach a broader market. Previous research has extensively analyzed the specialization dilemma faced by for-profit firms. We extend the analysis to knowledge-sharing groups in the marketplace of ideas. Using data on over 1,500 technology groups collected from an online event-organizing platform over a fifteen-year period, we measure the effect of topical focus, rarity, novelty, and technical exclusivity on audience growth, retention, and sustained engagement. We find that knowledge-sharing groups benefit marginally by specializing in rare topics but not in new topics. The strongest predictor of growth and survival is whether the group is associated with technically sophisticated topics, regardless of the breadth of focus, even though technical topics are less widely accessible. We conclude that what matters is not how specialized the organization, but how the organization is specialized.

## Introduction: The specialization dilemma

Organizations with finite resources confront a tradeoff between targeting investments of time, effort, and tangible resources to a particular market niche or diversifying investments more broadly [1]. Those that diversify have broader appeal and are better protected from risks within a particular market niche, but they are also more vulnerable to competition from specialists with concentrated expertise, economies of scale, and a more clearly differentiated identity that better conforms to the nuanced expectations of a narrowly targeted audience [2, 3]. Generalist organizations can provide high-bandwidth signals to stakeholders that focus on different aspects of expertise and performance, but a strategy that spans disparate categories can also impair market perception of product quality, organizational identity, technical expertise, and specialized leadership [4–8] compared to the specialist advantage in building a reputation with a more narrowly defined audience [9–11].

This specialization tradeoff has spurred extensive research on the performance of specialist and generalist organizational strategies. Previous studies have largely focused on the effect on the bottom line among firms in the for-profit sector [12–14]. For example, Toh and Kim [13] document how an organization’s technological specialization responds to uncertainty reflected in their stock-option volatilities. Similarly, Garcia-Vega [14] quantifies the effect of a firm’s R&D expenditures as a share of total sales.

The increasing importance of knowledge as an economic asset warrants greater attention to organizations that operate outside price-coordinated markets for tangible commodities [15]. The transition to a knowledge-based economy opens new opportunities for organizations whose primary mission is to generate, proliferate, and exchange information. Think tanks, public agencies, and universities play a significant role in facilitating the active exchange of knowledge and ideas and often provide a forum that allows people to share knowledge with others who differ in institutional and professional affiliations, age cohort, or ethnic and racial group [16]. This knowledge sharing can promote new technologies and enable future innovations, leading to the rewiring of networks of technologists and entrepreneurs in social exchanges that increase the likelihood of innovation and the founding of highly specialized start-up firms [17].

Although organizations in the knowledge economy generally do not compete in the for-profit marketplace for physical commodities, they compete with one another for attention in the marketplace for ideas and confront a similar strategic tradeoff between specialization and diversity. Their marketing strategies allow greater flexibility due to the jointness of supply and negligible marginal costs in the production and diffusion of knowledge [18, 19]. Although these groups are not typically motivated by financial gain, they nevertheless face a strategic decision as they compete to attract audience share; whether to focus effort and resources on highly specialized expertise or to appeal to a broader spectrum of the market [1, 20]. Specialists in the knowledge economy are characterized by a narrower focus and greater depth of knowledge while generalists are marked by access to more diverse knowledge.

As in the for-profit sector, each strategy has associated payoffs. Knowledge-sharing organizations may have better fitness when encountering unpredictable technological innovation by being diversified across multiple audience segments [21]. On the other hand, specialists that filter and curate information for a specific audience are more likely to gain a reputation for focused expertise and information quality [22]. Furthermore, while generalist groups with broad topical coverage may appeal to a broader audience, they risk having an amorphous identity. Studies in organizational identity have shown that audiences for information products like film and software may be put off by the ambiguity associated with generalist strategies [22–24]. In short, while case-specific examples have documented the advantages of both generalist and specialist strategies among knowledge-sharing groups outside the for-profit sector, it remains an empirical question whether a specialized focus is generally advantageous.

Our study makes two contributions to the literature on organizational specialization. First, using attendance records at self-organized high tech knowledge-sharing groups hosted on Meetup.com, we extend the analysis of specialist and generalist strategies to non-profit organizations competing in the marketplace of ideas. Second, we introduce three dimensions that qualitatively distinguish types of specialization that are especially relevant to nonprofit knowledge-sharing organizations. Previous research has focused extensively on the effects of *breadth* of focus. Our study also takes into account the *type* of focus: the rarity, novelty and technical exclusivity of topics around which Meetup groups specialize in their knowledge and information sharing. We also develop novel metrics to evaluate performance of knowledge-sharing organizations for which traditional financial measures of performance do not apply. We use these metrics to assess the relative effectiveness of strategies that differ in the extent and type of specialization.

To preview the results, we found that breadth of focus alone does not fully explain a knowledge-sharing group’s ability to attract and retain new members. Groups benefit marginally by specializing in rare topics but not in new topics. The strongest predictor of growth and survival among knowledge-sharing organizations is whether the group is associated with technically sophisticated topics, regardless of the breadth of focus. Our results show that the performance of knowledge-sharing groups depends not only on how specialized the organization is but also on how the organization is specialized.

### How specialized or how to specialize: Rarity, novelty, and exclusivity

Inconsistent results across case-specific studies of the specialization trade-off may reflect not only differences in firms and markets but also the sensitivity of the trade-off to the *type* of specialization. Simply put, in grappling with the strategic dilemma about *how focused to be*, the organization may also need to consider *how to be focused*. Our research is motivated by the possibility that the consequences of specialization–particularly in the marketplace of knowledge and ideas–may depend on whether the focus is on one of three dimensions that have received insufficient attention in previous studies: the rarity, novelty and technical exclusivity of the organization’s knowledge profile.

Research on “atypicality” broadly suggests that category spanners or otherwise non-conforming organizations–those that offer atypical products–tend to have inferior performance when evaluated by consumers [25, 26]. Organizations can be atypical within a generalist strategy by spanning multiple but disparate categories, while specialist organizations can also be atypical by focusing on a singular topic or a narrow spectrum of topics that are outside the mainstream. We disaggregated atypicality by distinguishing two distinct sources: the rarity of the topic (not widely encountered) and its novelty (not previously encountered). We measured the effects on performance of these two dimensions of atypicality, along with technical exclusivity and the degree of organizational specialization (i.e., breadth of focus).

Rarity refers to the number of organizations with a similar specialization. Rare topics and ideas are likely to be under-explored despite the opportunity for a breakthrough innovation [27, 28]. On the other hand, incorporating diverse and familiar topics can better explore the areas for which there is an untapped market and reveal potentially valuable opportunities for recombinant innovative activity through power in numbers. While focusing on commonplace topics may be helpful in attracting a large audience, unusual topics may be more effective at retaining audiences. If so, the outcomes of specialization may depend not only on the narrowness of focus but on the niche appeal of rare specialties.

Novelty refers to a topic that was not previously available. Uncharted, unexplored, and underexplored ideas may attract innovative technologists in the knowledge economy while generating niche appeal similar to that of rare topics. As with rare topics, groups organized around novel topics need to establish identity and legitimacy to overcome uncertainty related to unfamiliar ideas [29]. Although novelty and rarity pose similar challenges regarding identity and legitimacy, new topics are not always rare, and rare topics are often not new. For example, the computer mouse remained on the shelf at Xerox Park for years, waiting for the personal computer revolution to bring this old idea into widespread use. Conversely, novel ideas can experience sudden bursts in popularity that make them widely known in a short period of time, despite being in a formative state that remains to be fully developed. Unlike a rare idea that remains unknown to a broad audience for years or even decades, faddish new ideas may be widely circulated across the mainstream despite the absence of a proven track record or extensive development or testing. Examples of widely adopted novel ideas include the meteoric rise in popularity of blockchain technology and cryptocurrencies in the 2010s. The novelty of blockchain garnered initial skepticism and uncertainty that prevented wide acceptance in the marketplace for ideas, followed by a feeding frenzy of adoption that drove prices through the stratosphere, despite warnings that the technology was unproven and unregulated. Topic novelty thus captures a distinctive dimension of specialization that may affect the performance of a knowledge-sharing group.

While novelty and rarity characterize the extent of exposure to an idea across time and audience share, these attributes do not capture the dimension that is most directly associated with specialization: the *content* of an idea. Accordingly, we included an additional dimension, *technical exclusivity*, to measure the extent to which technically sophisticated content may restrict access to those who possess a sufficient level of training, skill, or other highly specialized knowledge required to effectively engage with a domain of information. Specializing in technically exclusive topics can serve as a gatekeeper, limiting the audience to those who can comprehend and/or appreciate information without the need for translation into more accessible language. Technical specialization allows an organization to attract an audience that is already well versed and shares a common language and skill set, even if the topics are neither rare nor novel. Technically exclusive knowledge can limit access to only a small niche with specialized training, but the prevalence of exclusive knowledge will depend on the distribution of skills and applications within a particular market. Exclusive content can be inaccessible, irrelevant, or otherwise unappealing to a broad or narrow audience, whether the knowledge is novel or familiar.

To sum up, we went beyond the traditional measure of the breadth of focus to test our hypothesis that the specialization trade-off depends not only on the number and diversity of topics offered by a knowledge-sharing organization, but also on how the topics are focused along three additional dimensions: rarity, novelty, and exclusivity. Using this multi-dimensional measure of specialization, we compared the outcomes for generalist and specialist strategies using data on knowledge-sharing organizations in the New York technology economy.

### Meetup.com and the New York technology economy

We compared the performance of specialist and generalist organizations in the knowledge economy using data obtained from Meetup.com, an online platform that facilitates networking events organized by groups whose members have shared interests. Meetup is a prominent example of self-organized groups on social media and networking platforms that have influenced public opinion on topics ranging from politics to science and technology [30–32].

Meetup.com was founded in 2002, shortly after the collapse of the dot-com bubble in 2000 and relatively early in the emerging technology economy of New York City. Over the ensuing two decades, the knowledge economy in New York grew to rival the leading hubs for technological entrepreneurship and innovation in the country. This development has been fueled in part by the rapid growth of technology-oriented Meetup groups [17]. Meetup provides a channel for interested individuals to form groups that organize events around topics of potential interest to other users. Every group on Meetup focuses on topics around which to organize and host events, and these events are explicitly organized to promote the exchange, diffusion, and evolution of knowledge and information among attendees and to facilitate “networking” among employers, job-seekers, and investors [33].

## Materials and methods

Data for this project were collected using Meetup.com’s API, in compliance with their license terms. All individual-level data collected from the API were anonymized. The research focuses on organizations (Meetup groups), not individual users, and the researchers have not interacted with or obtained any additional data from individual Meetup.com users. All analyses were based on data aggregated across time and across group members.

We used the self-identification and growth trajectories of over 1,500 technology related Meetup groups based in New York between 2005 and 2019 to assess the specialization trade-off among knowledge-sharing organizations in New York’s growing high-tech economy. Meetup groups tag themselves with descriptive topics that differentiate their identity and attract potential members with a shared interest. We collected data for over 3,300 unique topic tags with which to operationalize four dimensions of specialization: focus, rarity, novelty, and technical exclusivity.

**Fig 1:**
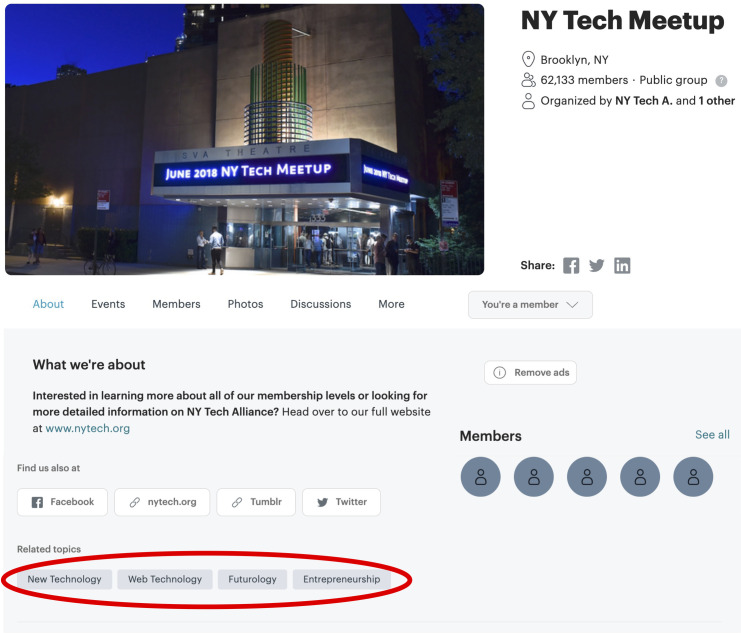
Topics listed by the NY Tech Meetup group. NY Tech Meetup hosts events bringing together interested technologists in the metropolitan region to share ideas related to the technology economy. Topic tags highlighted here are circled at the bottom of the figure.

The five most common topics in 2019 were “New Technology,” “Software Development,” “Web,” “Computer Programming,” and “Web Development.” Fig 1 shows the topic tags of the New York Tech Meetup group as an example.

Meetup groups promote knowledge spillover and sharing by hosting events organized around specialty topics and audiences. We used attendance at these events to measure group performance along four dimensions: relative size (number of members), growth (number of new members), retention rate (number of continuing members) and survival (number of active years). The construction of operational metrics for these four dimensions of performance, along with the four dimensions of specialization, are described in detail below.

### Focus: Specialization as narrowness of topic range

The conventional understanding of specialization typically refers to specialist organizations as those whose investments and products target a narrowly focused market segment [2, 34, 35]. Specialist Meetup groups focus on a small set of closely related topics while generalists cover a larger and more diverse range. Accordingly, our measure of focus accounts for both the number of topics and whether the topics are closely related, using weighted pairwise co-occurrences among all topics listed by each group. The more topics the group lists, the less likely that the topics are closely related and the less weight that we give to co-occurrences of topics by that group.

More precisely, for every topic, we constructed a weighted vector, ***x***, where each element corresponds to a Meetup group in the data set. Element *x*_*k*_ is weighted by $\log\left(\frac{N}{n_{k}}\right)$ if the *k*^*th*^ group has listed that topic, and 0 otherwise. *N* is the total number of topics across all groups, and *n*_*k*_ is the total number of topics listed by the *k*^*th*^ group. We calculated the cosine similarity between any two topics using their weighted vectors. Thus, a pair of topics listed together by a group with very few topics will have greater overall pairwise similarity than if the pair were to co-occur in groups with many topics. We then aggregated a group-level focus score by taking the average pairwise similarity across all possible pairs of topics listed by each group.

In addition to cosine similarity, we also measured focus using data on the number of redundant topics listed by a group. A group that lists redundant topics like “programming” and “coding” is presumably more focused than a group that lists two non-redundant topics (e.g., “programming” and “machine learning”). We manually aggregated all 3,412 topics listed across all groups into 1,779 distinct topic groups. We counted the number of non-redundant topics listed by each group, expressed as a proportion of all topics listed by the group. Redundancy is then scaled as one minus the proportion of non-redundant topics. For example, “3D graphics” and “3D animation” are considered part of the same topic group, and if a group lists “3D graphics” and “3D animation” as its only two topics, we would score this as 0.5 redundancy—redundancy is one minus the proportion: one distinct topic group over two total topics. A list of classified topics and distinct topic groups can be found in the S2 Data file that accompanies this paper.

The weighted cosine similarity score is significantly correlated with the rescaled redundancy measure (*r =* 0.270, *p <* 0.01). The significant correlation between pairwise similarity and redundancy suggests that both measures capture a latent dimension corresponding to the narrowness of topical focus. Both measures also address potential shortcomings of the other. The redundancy measure incorporates semantic interpretations of each individual topic, a qualitatively assessment that is prone to noise and with constrained variation, particularly for groups with only a small number of listed topics due to the small denominator in the proportion. In contrast, pairwise similarity is a quantitative measure of co-occurrences of all pairs of topics that does not involve subjective assessment and affords a higher level of granularity that can better discriminate among groups. The algorithm, however, is agnostic to the subjective meaning of topical content and considers only the number of times (and in which combinations) each topic appears in the data. In short, pairwise co-occurrence is only an indirect proxy for semantic similarity of the topics whereas the content of the topics was directly evaluated in the redundancy measure.

To leverage the close association between pairwise similarity and redundancy, we standardized and added the two scores as observable indicators of a single latent measure of the narrowness of focus. (We describe both measures in more detail and report separate results for each measure in the S1 Appendix file.) The narrower the focus, the greater the similarity and redundancy across the topics listed by each group, indicating greater specialization.

**Fig 2:**
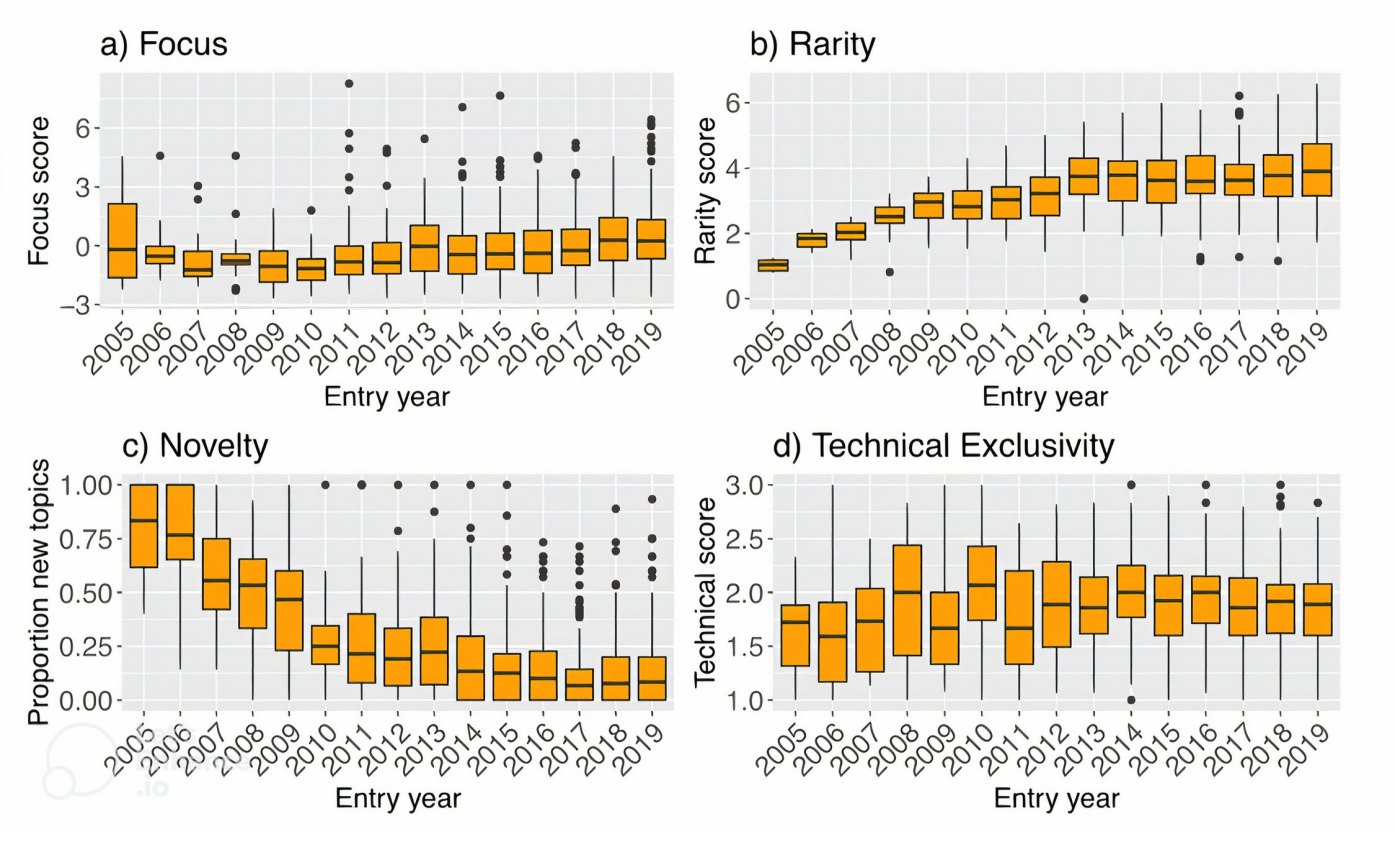
Trends over time along four dimensions of specialization. The distributions of (a) focus, (b) rarity, (c) novelty, and (d) technical exclusivity over time suggest that each measure captures a different dimension of specialization. Meetup groups trend towards associating with rarer topics, and the introduction of new topics declines over time.

Fig 2A shows the trend of the composite focus scores across Meetup groups founded between 2005 and 2019, and Table 1 shows the top five most and least focused groups as measured by the latent construct. Because the focus score is defined using the similarity between pairs of topics, this measure restricted the analysis to 1,504 groups that listed more than one topic. The five most focused groups have a clear identity within the broader technology economy: algorithmic trading, a popular code/text editor (vim), familiarizing seniors with technology and content creation for the YouTube platform. In contrast, the five least focused groups have either broad topics (e.g., innovation, social networking) or have pairs of topics that do not commonly co-occur within a single group (e.g., art and internet of things).

**Table 1:** Meetup groups with the most and least focused topics based on their choice of proximal topics.

***Most specialized***			
	**Group name**	**Focus**	**Sample Topics**
1.	Algorithmic Trading Factory	8.27	*Black-box trading education*, *Super-fast trading*
2.	The New York Vim Meetup	7.07	*Vim*, *Text editor*, *Learn vim*, *Vim plugins*
3.	Digital Grandparents	6.43	*Web*, *Seniors*, *Over 50*, *Active seniors*
4.	YouTubers NYC	6.22	*YouTube marketing*, *Online video creation*, *YouTube collaboration*
5.	YouTubers of NYC Meetup	6.10	*YouTube*, *Film and video production*, *YouTube marketing*, *YouTubers*
***Most generalized***			
	**Group name**	**Focus**	**Sample Topics**
1.	Manhattan Algorithmic Innovations	-2.70	*Artificial intelligence*, *Innovation*, *New technology*, *Startup businesses*
2.	NY eCommerce Mobile Retailers Meetup	-2.69	*Social networking*, *Mobile technology*, *E-Commerce*
3.	AWE Nite NYC	-2.68	*New media*, *Digital marketing*, *Augmented reality*
4.	Village Tech Breakfast	-2.68	*Futurology*, *Software development*, *Professional networking*, *Venture capital*
5.	NYC Dev	-2.62	*Art*, *New Technology*, *Music*, *Internet of things*, *Technology startups*, *Coders*

Among groups with comparable narrowness of focus, there is considerable variation in the *types* of knowledge. We hypothesize that performance outcomes will depend not only on overall topical focus but also on *how* the group is focused along the three dimensions of rarity, novelty, and technical exclusivity. The construction of these three measures is detailed below.

### Beyond focus: How are groups specialized?

#### Topic rarity

We measured rarity as the inverse frequency of each of a group’s topics relative to the overall distribution of topics across all groups in the year the group was founded. We first constructed an incidence matrix of groups and topic tags for each year and reweighted each element based on the TF-IDF weighting scheme from text mining. TF-IDF, or term-frequency/inverse document frequency, assigns greater weight to topic co-occurrences that are relatively rare (corresponding to “inverse document frequency”). For example, two documents that share a word that is only used in those two documents are more similar than two documents that share a word that is used in every document (making the pairwise co-occurrences entirely meaningless). In our data, topics are treated as “terms” and groups as “documents” such that rare topics in each year are given more weight while common topics that are shared by many groups are given less weight. We calculated a group’s topic rarity score as the average across all the TF-IDF weighted values. Groups will have higher topic rarity scores if they list topics that are rare relative to the entire pool of Meetup groups, each measured at the time of the group’s founding.

Note that the maximum rarity increases with the number of groups. A topic that only appears in one group out of 100 total groups will be scored with greater rarity than a topic that appears in one out of ten total groups. Fig 2B shows the distribution of group-level topic rarity scores between 2005 and 2019. The aggregate trend is increasing, reflecting the increase in the number of Meetup groups over time. To correct for this, we standardized rarity scores for each year to have zero mean and unit standard deviation. This removes the upward trend over time and makes rarity scores comparable between groups founded in different years. Table 2 shows the five groups with the most and least rare topics across the years they were active on the Meetup platform.

**Table 2:** Meetup groups with the most and least common topics across the years they were active.

***Rare topics***			
	**Group name**	**Rarity**	**Sample Topics**
1.	Digital Grandparents	6.489	*Web*, *Seniors*, *Over 50*, *Active seniors*
2.	SEO Mastermind NYC Meetup	6.320	*Search engine optimization*, *SEO business development*, *SEO training*
3.	NYC HoTT Group	6.228	*Functional programming*, *Category theory*, *Type systems*, *Model checking*
4.	YouTubers of NYC Meetup	6.214	*YouTube*, *Film and video production*, *YouTube marketing*, *YouTubers*
5.	Agritecture: Global Meetups	6.214	*Sustainable agriculture*, *Sustainable food*, *Agtech*, *Urban farming*
***Common topics***			
	**Group name**	**Rarity**	**Sample Topics**
1.	NYC Web Design Meetup	0.792	*Web design*, *Graphic design*, *New technology*, *Business strategy*
2.	NY Tech Meetup	0.805	*New technology*, *Web technology*, *Entrepreneurship*
3.	SQL NYC, The NoSQL & New SQL Database Big Data Meetup	1.003	*Big data*, *Open source*, *Software development*, *Data analytics*
4.	Graphic Design NYC	1.084	*Web development*, *Digital media*, *Creativity*, *New Technology*, *Artists*
5.	Silicon Alley: Tech Startup Community	1.187	*Internet professionals*, *Web technology*, *Mobile technology*

#### Topic novelty

We measured the level of novelty of a group’s topics by counting the number of new topics the group introduces and calculating the proportion of new topics at the time of the group’s founding. A Meetup group is considered to have introduced a new topic if one of the topics it lists at the time of its founding has not been previously introduced by another group in a previous year. A group’s topic novelty score is the proportion of new topics it lists that appear for the first time in the year of the group’s founding, out all of the topics that it lists. 18 groups between 2005 and 2019 have listed only topics that have not been previously used at the time of their founding (maximum novelty) while 492 groups listed only existing topics at the time of their founding (minimum novelty). Fig 2C shows the distribution of novelty scores by groups’ entry years. The proportion of new topics introduced by groups steadily declines over time. Although it is harder to be rare when there are few groups on Meetup, it is easier to be novel. Like the rarity scores, we standardized novelty scores for each year to have zero mean and unit standard deviation to make them comparable between groups founded in different years.

#### Technical exclusivity

We measured a topic’s technical exclusivity by manually classifying each topic into one of three levels: general interest topics (e.g., entrepreneurship, law, education), broad technology topics (e.g., robotics, big data, algorithms), and technically specialized topics (i.e., involving specific frameworks, software, or programming languages). Each topic was then assigned a score ranging from one for general interest to three for technically specialized. The classification was implemented by two independent raters, one of the authors and a research assistant familiar with the technology economy of New York. The two raters agreed on 73% of over 3,400 classifications (κ = 0.58), and the overall correlation between the ratings was 0.728. Each topic was then assigned the average of the two ratings and each group was assigned the average of the technical scores across all the group’s listed topics. Table 3 shows the five groups with the most and least technical topics, and Fig 2D reports the distribution of technical exclusivity scores of entering groups over time. As with the other three measures of specialization, technical exclusivity scores were normalized to have zero mean and unit standard deviation to facilitate comparison across specialization measures that lack an intuitive unit of measurement.

**Table 3:** Meetup groups with the most and least technical topics.

***Most technical***			
	**Group name**	**Tech Score**	**Sample Topics**
1.	NYC on Rails	3	*Ruby*, *Ruby on Rails*
2.	Socially Functional	3	*Functional programming*, *Scala*, *Haskell*, *Clojure*, *Erlang programming*
3.	Clojure NYC	3	*Functional programming*, *Clojure*
4.	NY Java Special Interest Group	3	*Java*, *NY Java developers*, *NYJavaSIG*
5.	jQuery NYC	3	*JQuery*, *JavaScript*, *CSS*
***Least technical***			
	**Group name**	**Tech Score**	**Sample Topics**
1.	StoryCode NYC	1	*Transmedia*, *New media*, *Web television*, *Film*
2.	Blogger Babes NYC & Tri-state	1	*Style bloggers*, *Lifestyle bloggers*, *Female entrepreneurs*, *Blog marketing*
3.	New York Education Tech Entrepreneurs	1	*Education entrepreneurs*, *Education and the cloud*, *Social media education*
4.	Aging 2.0	1	*Aging*, *Healthy aging*, *Innovation*, *Quantified self*
5.	Digital Grandparents	1	*Web*, *Seniors*, *Over 50*, *Active seniors*

### Correlations across the four specialization dimensions

**Fig 3:**
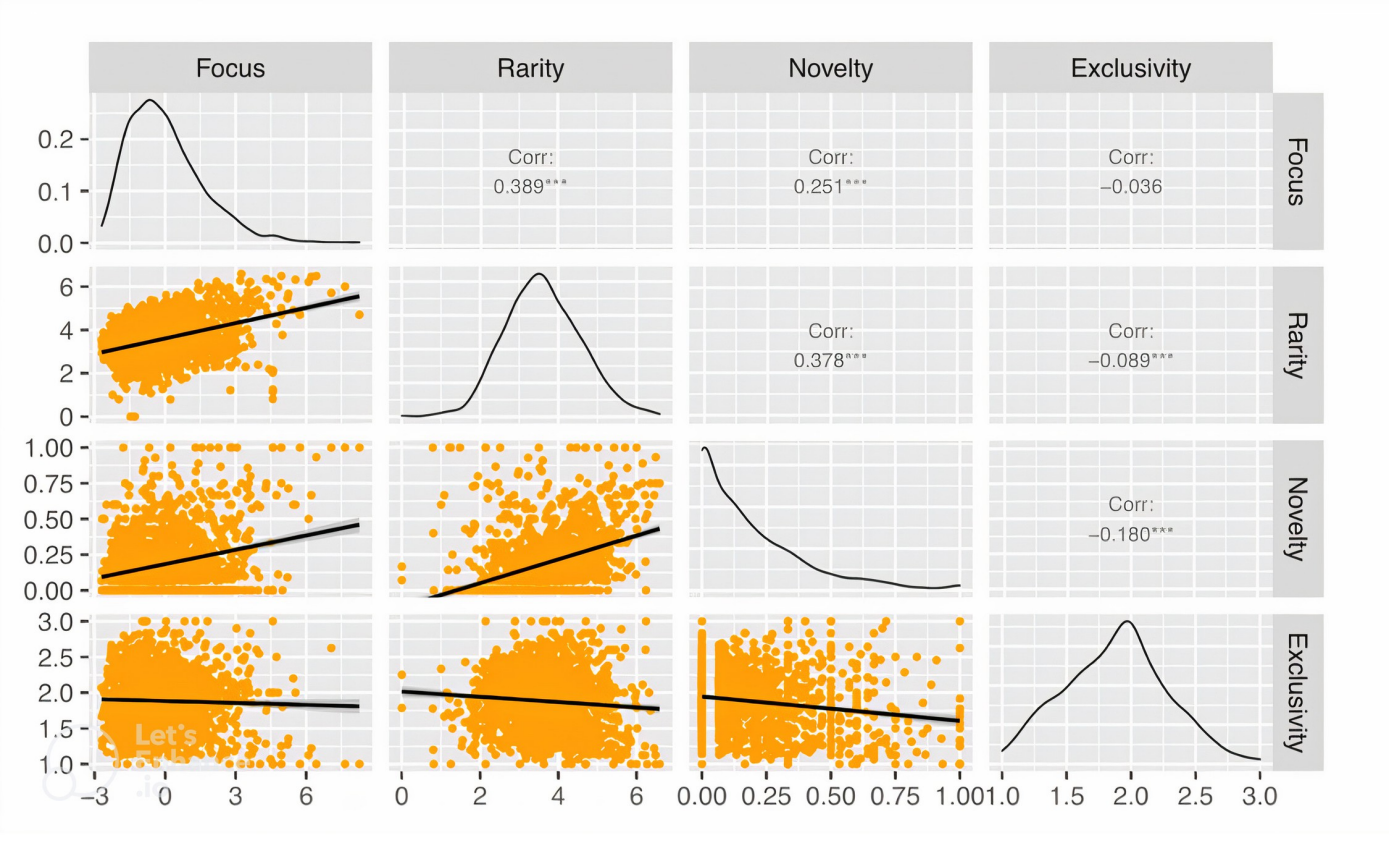
Pairwise correlations among four measures of specialization in the topic profiles of Meetup groups. Focus, rarity and novelty are positively correlated with one another but the pairwise correlations are relatively small. Tehnical exclusivity is negatively correlated with rarity and novelty.

The four measures of specialization–focus, rarity, novelty, and technical exclusivity–are expected to be correlated if they overlap in capturing a latent underlying dimension of specialization. Fig 3 shows the six pairwise correlations among the four measures, using Meetup groups (not topics) as the unit of analysis. Focus has a positive correlation with both rarity (*r =* 0.389, *p* < 0.01) and novelty (*r =* 0.251, *p* < 0.01), indicating that groups with narrowly focused topics tend to have rarer and/or newer topics. A group’s rarity and novelty scores have a positive correlation (*r* = 0.378, *p* < 0.01), and newer topics also tend to be less common. A group’s technical exclusivity score is negatively correlated with both its rarity (*r* = -0.089, *p* < 0.01) and novelty (*r =* -0.180, *p* < 0.01) scores, suggesting that knowledge-sharing groups with either rare or new topics tend not to be highly technical. Technical exclusivity is negatively correlated with focus, but the correlation is not significant at the 95% confidence level (*r* = -0.036, *p* = 0.168).

The overall low pairwise correlations among the four dimensions of specialization suggest that there is considerable heterogeneity in how knowledge groups specialize their topical content. For example, the groups NYC jQuery and NYC Crowdfunding both have focus scores above two (two standard deviations above the mean), but the technical exclusivity score for NYC jQuery is almost three times greater than that of NYC Crowdfunding. Similarly, while both the Full Stack Coding Meetup and the AlgoTrading Meetup have focus scores above two, all the topics associated with AlgoTrading were new at the time of its founding while none of the topics associated with Full Stack Coding were new. The different specialization dimensions captured by each of the four measures allow for a more granular analysis of performance among organizations with different specialization strategies.

### Measures of group performance

Among technology-related knowledge organizations, top-performing groups can be a powerful force in shaping the discourse on technological innovation. These groups do not create physical commodities for profit; rather, they bring people together to share and exchange ideas. Accordingly, evaluation of the performance of these groups requires measures other than what is typically used for organizational performance, such as financial returns, market share, or product launches. Knowledge organizations instead depend on proliferation and longevity of their associated ideas as well as the quantity and quality of the audience they attract. We therefore measured performance along four dimensions: audience share, as indicated by group size and growth, and participant retention and survival of the group, which measure the group’s ability to attract and sustain a dedicated and engaged audience. We hypothesize that these four outcome measures will be sensitive to whether and how the organization chooses to specialize. The construction of the four outcome measures is detailed below.

Average *size* of the group is measured as the log of the total number of unique individuals who attended an event hosted by the group each year, averaged across all years that the group was active. Maintaining a sizable audience is critical to survival of a knowledge group, and this measure of size indicates how well a group can attract an audience. Specialization may affect not only a group’s success in attracting an audience, but also its potential to *grow* its audience. A group’s audience will naturally fluctuate throughout the time that a group is active. The typical life cycle is for the number of people who attend a group’s events to start out small but to grow over time as the group matures. However, over the course of our 15-year observation period, many groups become inactive, and attendance often declines shortly before the group’s demise. An aggregate measure of attendance would miss the rise and fall over the group’s life span. Instead, we measure a group’s growth as the number of new members it attracts in a particular year who had not attended any Meetup events in the previous year. Each group is then scored with the proportion of the new-member cohort for a given year that attended an event hosted by that group, and the year-specific scores were then averaged across each of the years that the group was active. This growth measure is intuitively the percentage of new members to the Meetup knowledge network each year that a group attracted to its events. (Although the year-specific scores would be useful for a time-series analysis, these scores cannot be calculated for the specialization measures since Meetup groups rarely change their affiliated topics after the group is formed. Note also that the group “NY Tech Meetup” was excluded as an extreme outlier in the measure of new-member growth, as this was the dominant Meetup group in attracting new members between 2005 and 2010, with event attendance accounting for up to 75% of the new cohort in each of these years, compared to 4% for the group with the second highest percentage.)

Some knowledge organizations may value a dedicated audience for sustained knowledge exchange rather than a large audience that may cycle in and out of the group without having a sufficient opportunity to share ideas. We thus measure audience *retention* as the average proportion of people who attended a group’s events in the previous year who continued to attend the group’s events each year that the group was active. Out of the 1,504 groups in our sample, we could only calculate retention for 995 groups since this measure requires groups to have hosted events for at least two consecutive years.

Finally, these outcome measures are only relevant to a group insofar as they are actively hosting events. A group can have large audience, large new audience share, and/or high retention but still be short-lived. Given that the data span almost 15 years, we can observe the entire life span from the group’s first formation to its eventual state of inactivity. We define an inactive group as one did not host any events in the calendar year 2019, the year the data were collected. We used this measure, along with the date of the group’s first and last events, for a survival analysis that estimates the hazard of inactivity as an additional measure of performance.

Taken together, these four measures of performance capture both the quantity and quality of audience that a knowledge organization can engage. A highly successful knowledge organization is expected to have and retain a large and growing audience, along with a high likelihood of survival.

## Results

### Broader focus is generally associated with better performance

Table 4 reports coefficient estimates for cross-sectional linear regression models for the association between a group’s breadth of focus and its size (model 1), growth (model 2), audience retention (model 3), and hazard of inactivity (model 4). Controlling for group *i*’s entry year, the models include only one other predictor–narrowness of focus–for two reasons. First, focus comes closest to the conventional understanding of specialization. Second, the other three dimensions measure *how* the organization is focused, not how much. We therefore begin by including only focus as a measure of the *extent* of specialization, and then add the three measures of the *type* of specialization. The focus measure is standardized to have zero mean and unit variance, and all significance tests used robust standard errors.

**Table 4:** Linear model of group performance predicted by topic focus. Coefficient estimates of cross-sectional linear regression models for predicting a group’s size, growth, audience retention, and survival, using a group’s topic focus score as the lone predictor.

	*Dependent Variable*:			
	Avg. Num Attendees (log)	Avg. % New Cohort	Avg. % Retention	Hazard of Inactivity
	(1)	(2)	(3)	(4)
Entry year	-0.151**	-0.046**	0.122	0.193**
	(0.012)	(0.004)	(0.160)	(0.018)
Focus score	-0.137**	-0.011	-1.369**	0.005
	(0.041)	(0.007)	(0.527)	(0.043)
Observations	1,504	1,503	995	995
R^2^	0.105	0.166	0.008	

*p < 0.05

**p < 0.01

Models 1–3 use aggregate performance over all years from 2005 to 2019 (see Eq 1). We are limited to a cross-sectional analysis because the measure of specialization is not time varying. Groups are associated with one set of topics throughout the entire observation period, hence the breadth of focus is invariant.


$$\;Performance_{i}\;=\;\beta_{0}\;+\;\beta_{1}\;EntryYear{\;}_{i}\;+\;\beta_{2}\;Focus_{i}\;+\;u_{i}$$ (1)

Although topical focus is invariant, a group’s survival captures a time-varying measure of performance. Model 4 shows coefficient estimates of a Cox proportional hazards model for the hazard of a group becoming inactive. The baseline time for each group is the day it held its first meetup event; thus, the time horizon is a measure of the age (in days) of the group rather than absolute time. The modeling equation for the Cox proportional hazards model is given in Eq 2, where *h*(*t*) is the hazard function of a group becoming inactive, and *t*_0_ is the year the group was founded.


$$h_{i}(t)\;=\;h\left(t_{0}\right)\;\text{exp}\;\left(\beta_{0}\;+\;\beta_{1}\;EntryYear_{i}\;+\;\beta_{2}\;Focus_{i}\right)$$ (2)

Across the years that a group was active, controlling for the year that the group was founded, a narrow focus is associated with a smaller audience and lower audience retention. A one standard deviation increase in narrowness of focus is associated with a 13.7%-point decrease in event attendees (*p* < 0.01) and a decrease of 1.4 points in the percentage of retained audiences (*p* < 0.01). These results suggest that the level of a group’s specialization, as measured by narrowness of topical focus, has a negative effect on performance. While largely independent of a group’s survival and growth, highly focused groups attract a smaller audience and have a harder time retaining it.

For the survival model, a global test of the proportional hazards assumption yields a *p*-value of 0.09, and we fail to reject the null hypothesis that the proportional hazards assumption holds. An individual test of the entry year variable is marginally significant (*p* = 0.049) for rejecting the proportional hazards assumption, which is also driving the global test result. (We further explore possible time-dependent specifications for the survival model in the S1 Appendix file, but our alternate models do not substantially depart from the results reported in Table 4.)

### Specialization promotes performance when focused on topics with technical exclusivity

Table 5 reports results for the same four performance outcomes but includes three additional measures of specialization as predictors (see Eqs 3 and 4). *Score*_*ij*_ in the equations represents the sum over the three additional dimensions of specialization, where *j = 1*, *2*, *3* indexes the rarity, novelty, and exclusivity scores. All specialization scores are standardized, and robust standard errors are used for each model. The four outcome measures are identical to those in Table 4. As in Table 4, a test of the proportional hazards assumption for the survival model fails to reject the null hypothesis that the proportional hazards assumption is satisfied (*p* = 0.128).


$$\;Performance_{i}\;=\;\beta_{0}\;+\;\beta_{1}\;EntryYear_{i}\;+\;\beta_{2}\;Focus_{i}\;+\;\sum_{j=1}^{3}\beta_{2+j}\;Score_{ij}\;+\;\sum_{j=1}^{3}\beta_{5+j}\;Focus_{i}\;\times \;\;Score_{ij}\;+\;u_{i}$$ (3)

$$h_{i}(t)\;=\;h\left(t_{0}\right)\text{exp}\left(\beta_{0}\;+\;\beta_{1}\;EntryYear_{i}\;+\;\beta_{2}\;Focus_{i}\;+\;\sum_{j=1}^{3}\beta_{2+j}\;Score_{ij}\right.\left.\;+\;\sum_{j=1}^{3}\beta_{5+j}\;Focus_{i}\;\times \;\;Score_{ij}\right)$$ (4)

**Table 5:** Cross-sectional linear regression model of group performance predicted by multiple dimensions of specialization. The table reports coefficient estimates for predicting a group’s size, growth retention, and survival using a group’s topic focus, rarity, novelty and technical exclusivity scores.

	*Dependent Variable*:			
	Avg. Num Attendees (log)	Avg. % New Cohort	Avg. % Retention	Hazard of Inactivity
	(1)	(2)	(3)	(4)
Entry year	-0.170**	-0.032**	-0.408	0.179**
	(0.018)	(0.004)	(0.247)	(0.023)
Focus score	-0.002	-0.012	-1.645**	-0.022
	(0.045)	(0.010)	(0.569)	(0.051)
Rarity score	-0.105	-0.086**	1.984**	0.093
	(0.054)	(0.016)	(0.711)	(0.065)
Novelty score	-0.163**	0.030	-0.927	-0.048
	(0.056)	(0.019)	(0.652)	(0.063)
Exclusivity score	0.241**	0.008	4.311**	-0.140**
	(0.038)	(0.009)	(0.441)	(0.041)
Rarity × Focus	-0.038	0.024*	-0.724	-0.010
	(0.037)	(0.011)	(0.529)	(0.043)
Novelty × Focus	-0.031	-0.027**	-0.406	0.025
	(0.033)	(0.009)	(0.352)	(0.035)
Exclusivity × Focus	-0.010	0.002	0.121	0.024
	(0.037)	(0.007)	(0.412)	(0.037)
Observations	1,504	1,503	995	995
R^2^	0.160	0.206	0.107	

*p < 0.05

**p < 0.01

The four models test 1) the marginal effect of specializing on topics that are rare, novel, or technical, over and above the narrowness of focus, and 2) the interaction between focus and each of the three types of specialization. Table 5 shows that controlling for the year the group was founded, the coefficient for focus is no longer statistically significant at the 0.05 level for any of the outcomes other than retention (model 3), where it continues to be negative (*β* = -1.65, *p* < 0.01). In other words, the bivariate associations between narrowness of focus and the four measures of performance reported in Table 4 are largely mediated by the type of focus, as indicated by the decrease in the coefficient for overall topical focus when the three additional measures of the type of specialization are included in the model.

Novelty has a negative association with the number of event attendees, and groups that are highly focused on new topics also experience inhibited growth. A one standard deviation increase in a group’s topic novelty score is associated with a 16.3 percentage point decrease (*p <* 0.01) in average attendees at the group’s events. In terms of growth, the influence of novelty depends on the level of focus of the group’s topics. Highly focused groups with highly novel topics attract fewer new participants than unfocused (generalist) groups with novel topics (*β* = -0.027, *p* < 0.01). A group that is centered around new topics tends to be smaller, and its growth trajectory depends on whether the group also focuses on those new topics. Focusing narrowly on new topics appears to hinder a group’s growth, while focusing more broadly on new topics confers an advantage.

Rarity is associated with weaker attraction for new cohorts (*β* = -0.086, *p* < 0.01), although this negative effect is greatly attenuated if rarity is associated with a narrower focus, as indicated by the interaction coefficient (*β* = 0.024, *p* < 0.05). Groups with rarer topics also experience higher retention rates; a one standard deviation increase in rarity is associated with a 1.98%-point increase in the group’s average audience retention rate (*p <* 0.01).

Technical exclusivity is associated with improved performance on three of the four outcome measures: logged event attendees (*β* = 0.248, *p* < 0.01), retention of new members (*β* = 4.311, *p* < 0.01) and lower hazard of falling to inactivity (*β* = -0.160, *p* < 0.01). However, the interactions with focus are not significant, which means the positive associations do not depend on the group’s overall topical focus.

The two most important takeaways from Table 5 are (1) most of the bivariate association between performance and breadth of focus is mediated by the type of focus (assuming that the level of specialization is causally prior to the type), and (2) organizing a Meetup group around highly technical topics has the potential to attract a larger audience and retain them for a longer period.

## Discussion

Organizations competing for market share confront a strategic dilemma: whether to narrowly specialize and thereby dominate a particular niche or to diversify and appeal to a broader range of interests. Previous research has investigated the specialization trade-off among for-profit firms. Our study extends the analysis to high-tech knowledge-sharing organizations in the non-profit sector. The growth of the knowledge economy continues to spur new organizational forms that depart from the traditional for-profit firm. This paper focuses on the performance of knowledge organizations and the unique strategic challenges of differentiating and establishing an identity in the marketplace for knowledge and ideas. While these organizations do not directly produce tangible products that can be price-coordinated, they nevertheless compete with one another for attention and audience share, and their survival depends on continued engagement with knowledge workers seeking to share and exchange new ideas.

Our study leverages a unique data set from Meetup.com, an on-line platform that hosts numerous knowledge-sharing groups with varying organizational structure and topical focus, all of whom organize events for the purpose of attracting an interested audience. Using 15 years of data on knowledge-sharing groups in New York’s burgeoning technology economy, we found that the narrowness of topical focus does not fully capture the relationship between specialization and organizational performance. Three additional dimensions–rarity, novelty, and exclusivity–reveal a fuller and more nuanced understanding of the strategic dilemma.

Our study adds additional insight by measuring performance along multiple dimensions: a group’s size, growth, audience retention, and survival. A narrow focus limits performance on three of the four outcome measures but not the likelihood of survival. We found that survival depends primarily on whether the organization is associated with technically exclusive topics, regardless of the overall breadth of focus. Moreover, the effect of topical focus on performance is largely mediated by *how* the group is focused. A focused strategy only performs better if the organization specializes on a rare or highly technical topic, especially for attracting new members. In contrast, focusing on novel topics inhibits growth in the marketplace of ideas.

Despite the richness and granularity afforded by these data, our study has several limitations. First, our results hinge on the assumption that Meetup groups are competing with one another in the marketplace for ideas and that there is market demand for the knowledge that is offered and exchanged. It is important to acknowledge that Meetup groups may be formed for a variety of reasons, and organizers may not know the mix of specialization of groups outside their niche; nor are they likely to regard audience participation, retention, or longevity as their primary motivation for forming the group. In short, our study relies on behavioral measures of specialization and performance, about which the group’s organizers may be unaware. Future research is needed to drill down into the subjective perceptions, intentions, and motivations that drive the behavioral measures.

Second, our survival measure considers a group to be inactive if it did not host any events in the calendar year 2019, the final year for which data were collected. About 10% of Meetup groups that had taken a hiatus of at least a year later became reactivated. It is therefore possible that groups coded as inactive might have reactivated after our data were collected in 2019. However, our data collection occurred shortly before strict public health limitations were placed on in-person gatherings, making it unlikely that groups that were inactive in 2019 would reactivate in 2020. Moreover, we do not observe any qualitative differences in the topic specializations of groups that took a one-year break and those that were continuously active; hence the results of our survival analysis are unlikely to change if groups coded as inactive had reactivated post-2019.

Third, inactive Meetup groups can rebrand themselves as a new group that focuses on new topics while retaining their pre-existing audience, and our performance measures would treat the old and new groups as distinct entities. However, rebranding a group with a different topical menu can also be regarded as the entry into the market of a new group, whose performance outcomes can be used to measure the association between specialization and performance, even if the standard errors are under-estimated due to the lack of complete statistical independence.

Lastly, our study is restricted to Meetup groups whose interests relate to technology, but these groups may also organize events around a broad range of topics beyond technology. Our findings should not be generalized beyond groups engaged in technical knowledge sharing, and future research could use these same Meetup data to explore and uncover more nuanced effects of specialization in other cultural domains, from sports and music to film and fashion.

In closing, the take-home message is two-fold. First, technical specialization pays off in a knowledge economy. Second, in the marketplace of ideas, there is more to specialization than narrowness of focus, the conventional framing in the organizational literature. Our study points to the need for a more nuanced, multi-dimensional analysis of how knowledge can be organized to better facilitate sharing and exchange. The catch-phrase is ironic: research on specialization has been too narrowly focused. What matters is not just how specialized is the organization, but also how the organization is specialized.

## Supporting information

S1 Data RData file with data frames all analyses. Curated data frame for components of the focus score calculation named focustfidf. Curated data frame for main regressions named groupstatic. Curated data frame for survival analysis named survdata in the data file.
(ZIP)

S2 Data CSV file with researcher categorization of Meetup topic labels into distinct topic groups. The file contains 2 columns where ‘Topic’ lists all individual Meetup topic labels that were observed in the data, and ‘Topic Group Identifier’ lists the corresponding ID of the topic group. Topics that are classified as belonging to the same topic group share the same ID.
(CSV)

S1 Appendix Technical appendix that discusses alternative specifications of focus and robustness check of the Cox proportional hazards assumption for survival models. (DOCX)
