# Supplementary material for: Specialization in the marketplace for ideas
Source: PLoS One. 2023 Oct 25;18(10):e0293355. doi: 10.1371/journal.pone.0293355 (PMC10599521; doi:10.1371/journal.pone.0293355)
Supplement: S1 Appendix — (DOCX) [file pone.0293355.s003.docx]

**APPENDIX**

**A.1. Alternative specifications of focus**

We used a latent construct of narrowness of focus as a composite measure of two highly correlated observable attributes: pairwise topical similarity and topic redundancy. Each observable measure addresses a potential shortcoming of the other. We show in this section that our main findings do not qualitatively change if we replace the composite with each of its constituent measures.

Tables A1 and A2 report coefficient estimates for models corresponding to the models in Table 4 and Table 5, respectively, except that the composite is replaced pairwise co-occurrence similarity among the topics listed by each Meetup group (A1) and topic redundancy (A2). Topic similarity is a negative predictor for audience size (*β =* -0.196, *p*<0.0$1$) and growth (*β =* -0.023, *p* < 0.01) and is not significant in predicting retention or survival. The coefficients for the effects on group size and growth are reduced while focus becomes a significant negative predictor for retention. Higher technical exclusivity predicts audience size (*β =* 0.025, *p*<0.0$1$), retention (*β =* 4.304, *p* < 0.01) and lower hazard of inactivity (*β =* -0.145, *p <* 0.01). Rare topics predict smaller group size (*β =* -0.108, *p <* 0.05), slower growth (*β =* -0.088, *p <* 0.01), and higher retention (*β =* 1.754, *p <* 0.05). New topics are associated with smaller group size (*β =* -0.153, *p <* 0.01). While rare topics indicate slower growth regardless of overall topical focus, focusing on rare topics attenuates the coefficient, with a positive interaction effect (*β =* 0.031, *p <* 0.01). In contrast, the interaction between focus and novelty shows that focusing only on highly novel topics inhibits growth (*β =* -0.025, *p <* 0.01).

**Table A1. Linear model of group performance using co-occurrence similarity in place of the composite measure of topical focus.** Coefficient estimates of cross-sectional linear regression models for a group size, growth and audience retention, with topical focus measured as the standardized pairwise co-occurrence similarity across the topics listed by each group.

|  | *Dependent Variable:* | | | |
| --- | --- | --- | --- | --- |
|  | Avg. Num Attendees (log)  (1) | Avg. % New Cohort  (2) | Avg. % Retention  (3) | Hazard of  Inactivity  (4) |
| Entry year | -0.153**  (0.012) | -0.046**  (0.004) | 0.064  (0.159) | 0.193**  (0.018) |
| Focus score (Avg. pairwise similarity) | -0.196**  (0.042) | -0.023**  (0.007) | -0.915  (0.636) | 0.028  (0.046) |
| Observations | 1,504 | 1,503 | 995 | 995 |
| R^2^ | 0.111 | 0.169 | 0.003 |  |
| **p*<0.05; ***p*<0.01 | | | | |

**Table A2. Linear model of group performance using co-occurrence similarity in place of the composite measure of topical focus, along with three additional measures of specialization.** Coefficient estimates of cross-sectional linear regression models for a group size, growth, and audience retention, with topical focus measured as the standardized pairwise co-occurrence similarity across the topics listed by each group, along with three additional measures of specialization: rarity, novelty and technical exclusivity. Topical focus is measured as the standardized pairwise co-occurrence similarity across the topics listed by each group.

|  | *Dependent Variable:* | | | |
| --- | --- | --- | --- | --- |
|  | Avg. Num Attendees (log)  (1) | Avg. % New Cohort  (2) | Avg. % Retention  (3) | Hazard of  Inactivity  (4) |
| Entry year | -0.166**  (0.018) | -0.032**  (0.004) | -0.409  (0.247) | 0.176**  (0.024) |
| Focus score | -0.055  (0.051) | -0.003  (0.010) | -0.974  (0.738) | 0.013  (0.060) |
| Rarity score | -0.108*  (0.055) | -0.088**  (0.016) | 1.754*  (0.714) | 0.094  (0.064) |
| Novelty score | -0.153**  (0.059) | 0.033  (0.019) | -0.833  (0.640) | -0.064  (0.066) |
| Exclusivity score | 0.250**  (0.038) | 0.009  (0.009) | 4.304**  (0.432) | -0.145**  (0.041) |
| Rarity $\times$ Focus | -0.001  (0.035) | 0.031**  (0.010) | -0.315  (0.593) | -0.005  (0.047) |
| Novelty $\times$ Focus | -0.021  (0.028) | -0.025**  (0.009) | -0.057  (0.428) | 0.030  (0.034) |
| Exclusivity $\times$ Focus | -0.009  (0.033) | 0.001  (0.006) | -0.350  (0.470) | -0.005  (0.039) |
| Observations | 1,504 | 1,503 | 995 | 995 |
| R^2^ | 0.159 | 0.206 | 0.100 |  |
| **p*<0.05; ****p*<0.01 | | | | |

Tables A3 and A4 report estimated coefficients for corresponding models in which focus is measured as redundancy (one minus the proportion of redundant topics listed by each Meetup group). Redundancy is a negative predictor of retention (*β =* -1.344, *p <* 0.01) in the bivariate analysis and is no longer a significant predictor for any of the outcome variables when the controlling for other dimensions of specialization. Rare topics continue to be negatively associated with size (*β =* -0.121, *p <* 0.05) and growth (*β =* -0.083, *p <* 0.01) while positively associated with retention (*β =* 1.937, *p <* 0.01). New topics continue to be associated with smaller group size (*β =* -0.186, *p <* 0.01), and technical exclusivity continues to be associated with better performance outcomes: group size (*β =* 0.241, *p <* 0.01), retention (*β =* 4.377, *p <* 0.01) and hazard of inactivity (*β =* -0.131, *p <* 0.01). There are no longer any significant interaction effects between focus and any of the other dimensions of topic choice. In short, the estimates of the main effects using redundancy as the measure of topical focus are consistent with those estimated from models using pairwise co-occurrence similarity. Together, these results are also qualitatively similar to the results presented in the main text using a composite measure of topical focus.

**Table A3. Linear model of group performance using topic redundancy in place of the composite measure of topical focus.** Coefficient estimates of cross-sectional linear regression models for group size, growth and audience retention, with topical focus measured as the proportion of a group’s redundant topics (standardized).

|  | *Dependent Variable:* | | | |
| --- | --- | --- | --- | --- |
|  | Avg. Num Attendees (log)  (1) | Avg. % New Cohort  (2) | Avg. % Retention  (3) | Hazard of  Inactivity  (4) |
| Entry year | -0.157**  (0.013) | -0.047**  (0.004) | 0.123  (0.160) | 0.195**  (0.018) |
| Focus score (Prop. redundant topics) | -0.048  (0.041) | 0.002  (0.007) | -1.344**  (0.505) | -0.017  (0.043) |
| Observations | 1,504 | 1,503 | 995 | 995 |
| R^2^ | 0.099 | 0.098 | 0.006 |  |
| **p*<0.05; ***p*<0.01 | | | | |

**Table A4. Linear model of group performance using topic redundancy in place of the composite measure of topical focus, along with three additional measures of specialization.** Coefficient estimates of cross-sectional linear regression models for group size, growth, and audience retention, with topical focus measured as the proportion of a group’s redundant topics (standardized).

|  | *Dependent Variable:* | | | |
| --- | --- | --- | --- | --- |
|  | Avg. Num Attendees (log)  (1) | Avg. % New Cohort  (2) | Avg. % Retention  (3) | Hazard of  Inactivity  (4) |
| Entry year | -0.173**  (0.018) | -0.034**  (0.004) | -0.475  (0.247) | 0.181  (0.023) |
| Focus score | -0.037  (0.051) | 0.018  (0.010) | -1.630**  (0.523) | -0.041  (0.046) |
| Rarity score | -0.121*  (0.054) | -0.083**  (0.016) | 1.937**  (0.717) | 0.101  (0.064) |
| Novelty score | -0.186**  (0.053) | 0.023  (0.016) | -1.220  (0.646) | -0.046  (0.060) |
| Exclusivity | 0.241**  (0.038) | 0.008  (0.009) | 4.377**  (0.450) | -0.131**  (0.041) |
| Rarity $\times$ Focus | -0.061  (0.039) | 0.008  (0.009) | -0.810  0.542 | 0.024  (0.044) |
| Novelty $\times$ Focus | -0.035  (0.036) | -0.020  (0.010) | 0.773  (0.435) | 0.007  (0.040) |
| Exclusivity $\times$ Focus | -0.005  (0.037) | 0.003  (0.008) | 0.458  (0.450) | 0.054  (0.039) |
| Observations | 1,504 | 1,503 | 995 | 995 |
| R^2^ | 0.161 | 0.203 | 0.109 |  |
| **p*<0.05; ****p*<0.01 | | | | |

**A.2. Cox proportional hazards assumption for survival models**

We use a Cox proportional hazards model to test for the effect of focus and topic choice on the survival of a Meetup group. This model estimates the coefficients associated with each predictor in its relationship to the hazard of a group being inactive (i.e., no longer hosting any events for one calendar year). The Cox proportional hazards model assumes proportional hazards relative to a baseline hazard, that is, a unit increase in a predictor must be associated with a multiplicative increase in hazard rate *for all values of time*. This assumption can be explicitly tested, and a violation of the proportional hazards assumptions suggests that a more refined model is needed such as one that incorporates predictors or effects that change with time.

Table A5 shows the results of a test of proportionality for the bivariate and multivariate models where the null hypothesis is that the proportional hazards assumption is satisfied. The global tests for both the bivariate and multivariate survival models fail to reject the null hypothesis at the 95% significance level. However, the coefficient for the entry year variable in both models and the technical score variable is not statistically significant at the 5% level. We further examined these measures by plotting the Schoenfeld residuals over time for a visual inspection of a discernable trend over time. Figure A1 shows the Schoenfeld residuals over time for the predictors for which we may reject the null hypothesis assumption of proportional hazards. Visually, there does not appear to be a trend exhibited by the residuals over time. We also fitted an extended Cox model with time varying coefficients, using the flexible functional form $\beta\left( t \right)=x_{0}+x_{1}\log(t+1)$ for the coefficient of entry year (both models) and technical score (multivariate model). For all of the models with this specification of the time varying coefficient, the estimated $x_{1}$ term is not statistically different from 0, thus favoring a time-constant coefficient. Results for this analysis can be made available upon request. Since the global test of the proportional hazards assumption suggests that the assumption cannot be reasonably rejected at the 95% confidence level, and the individual tests do not present any apparent time dependencies, in the main text, we only report the simple Cox proportional hazards model for estimating the effect of the predictors on the hazard of inactivity for a Meetup group.

**Table A5. Test for proportional hazards for the bivariate and multivariate models for the hazard of a Meetup group becoming inactive.** The null hypothesis for each test is that the proportional hazards assumption is satisfied, and the Cox proportional hazards model is appropriated specified.

|  | ***Bivariate Model*** | | |
| --- | --- | --- | --- |
|  | $\boldsymbol{\chi}^{\boldsymbol{2}}$**-statistc** | **Degrees of freedom** | ***p-*value** |
| Entry year | 3.869 | 1 | 0.049* |
| Focus score | 0.429 | 1 | 0.512 |
| GLOBAL | 4.748 | 2 | 0.093 |
|  | ***Multivariate Model*** | | |
|  | $\boldsymbol{\chi}^{\boldsymbol{2}}$**-statistc** | **Degrees of freedom** | ***p*-value** |
| Entry year | 5.075 | 1 | 0.024* |
| Focus score | 0.221 | 1 | 0.639 |
| Rarity score | 0.373 | 1 | 0.541 |
| Novelty score | 3.822 | 1 | 0.051 |
| Exclusivity score | 4.457 | 1 | 0.035* |
| Rarity $\times$ Focus | 0.617 | 1 | 0432 |
| Novelty $\times$ Focus | 0.196 | 1 | 0.658 |
| Exclusivity $\times$ Focus | 1.062 | 1 | 0.303 |
| GLOBAL | 12.566 | 8 | 0.128 |
| **p*<0.05; ***p*<0.01 | | | |


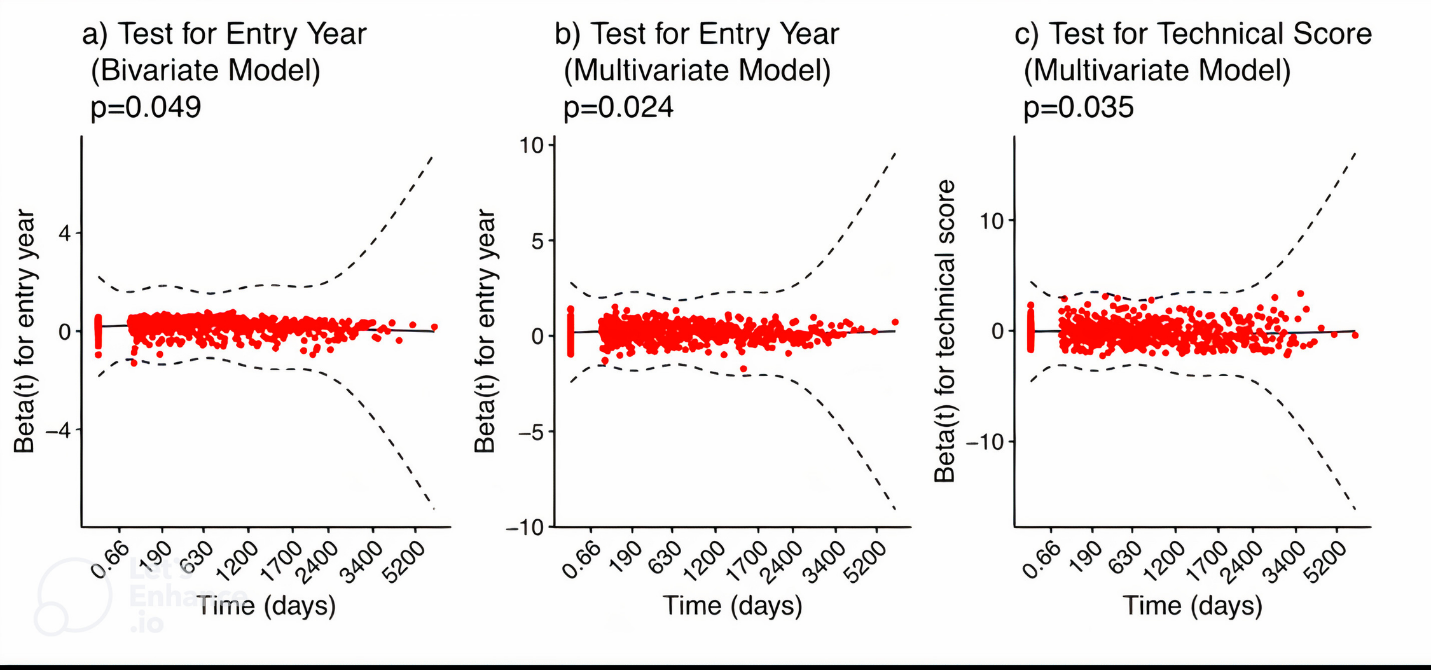


**[**Fig S4 about here; uploaded separately]

**Fig S4. Schoenfeld residuals over time in days (logged axis).** Schoenfeld residuals for a) the entry year variable in the bivariate model, b) the entry year variable in the multivariate model, and c) the technical exclusivity score variable in the multivariate model.
